# Supplementary material for: Identification of Freezing-Responsive microRNAs and Their Targets in Chinese Jujube by Small RNA and Degradome Sequencing
Source: Int J Mol Sci. 2026 Sep 4;27(17):7912. doi: 10.3390/ijms27177912 (PMC13566890; doi:10.3390/ijms27177912)
Supplement: Supplementary file 1 [file ijms-27-07912-s001.zip › Supplementary Material S4.pdf]

Table S4. Statistics of differentially expressed miRNAs in Jujube tree

Number	miRNA	mature_sequence	GenomeID
1	bcy-miR156	TTTGACAGAAGATAGAGAGCAC	NC_029683.1
2	bgy-miR156	TTTGACAGAAGATAGAGAGCAC	NC_029683.1
3	cme-miR156a	TGACAGAAGAGAGTGAGCAC	NC_029684.1
4	cpa-miR156e	TTGACAGAAGATAGAGAGCAC	NC_029682.1
5	csi-miR159c-3p	CTTGGACTGAAGGGAGCTCCC	NC_029689.1
6	ctr-miR319	TTGGACTGAAGGGAGCTCCC	NC_029689.1
7	eun-miR162-3p	TCGATAAACCTCTGCATCCAG	NC_029683.1
8	fve-miR156g-3p	GCTCTCTATGCTTCTGTCATC	NC_029683.1
9	fve-miR156g-5p	TTGACAGAAGATAGAGAGCAC	NC_029683.1
10	fve-miR162-3p	TCGATAAACCTCTGCATCCAG	NC_029683.1
11	gma-miR162c	TCGATAAACCTCTGCATCCAG	NC_029683.1
12	gma-miR319a	TTGGACTGAAGGGAGCTCCC	NC_029689.1
13	gma-miR319m	TTGGACTGAAGGGAGCTCCCT	NC_029680.1
14	gma-miR5368	GGACAGTCTCAGGTAGACA	NC_029689.1
15	gra-miR157a	TTGACAGAAGATAGAGAGCAC	NC_029683.1
16	gra-miR157b	TTGACAGAAGATAGAGAGCAC	NC_029683.1
17	hbr-miR156	TTGACAGAAGATAGAGAGC	NC_029683.1
18	hbr-miR159a	TTTGGATTGAAGGGAGCTCTA	NW_015454482.1
19	hbr-miR319	TTGGACTGAAGGGAGCTCCCT	NC_029689.1
20	hbr-miR6483	TATTGTAGAAATTTTCAGGATC	NW_015453418.1
21	hbr-miR6485	TAGGATGTAGAAGAGCATAA	NW_015453507.1
22	lja-miR167	TGAAGCTGCCAGCATGATCTG	NC_029680.1
23	lus-miR156h	TTGACAGAAGATAGAGAGCAC	NC_029682.1
24	mdm-miR156b	TGACAGAAGAGAGTGAGCAC	NW_015453542.1
25	mdm-miR156i	TGACAGAAGAGAGTGAGCAC	NW_015453542.1
26	mdm-miR162a	TCGATAAACCTCTGCATCCAG	NC_029683.1
27	mdm-miR162b	TCGATAAACCTCTGCATCCAG	NC_029683.1
28	mdm-miR482a-5p	AGGAATGGGCTGTTTGGGAAGA	NC_029682.1
29	mes-miR156c	TGACAGAAGAGAGTGAGCAC	NW_015453542.1
30	mes-miR156g	TGACAGAAGAGAGTGAGCAC	NW_015453542.1
31	mes-miR156h	TTGACAGAAGATAGAGAGCAC	NC_029683.1
32	mes-miR156i	TTGACAGAAGATAGAGAGCAC	NC_029683.1
33	mes-miR156j	TTGACAGAAGATAGAGAGCAC	NC_029683.1
34	mes-miR162	TCGATAAACCTCTGCATCCAG	NC_029683.1
35	mtr-miR156b-5p	TGACAGAAGAGAGTGAGCAC	NC_029684.1
36	mtr-miR319b-3p	TTGGACTGAAGGGAGCTCCC	NC_029689.1
37	nta-miR319b	TTGGACTGAAGGGAGCTCCCT	NC_029680.1
38	ppe-miR156g	TTGACAGAAGATAGAGAGCAC	NC_029683.1
39	ptc-miR156g	TTGACAGAAGATAGAGAGCAC	NC_029682.1
40	ptc-miR156i	TTGACAGAAGATAGAGAGCAC	NC_029683.1
41	ptc-miR156j	TTGACAGAAGATAGAGAGCAC	NC_029683.1

42	ptc-miR319a	TTGGACTGAAGGGAGCTCCC	NC_029689.1
43	ptc-miR319c	TTGGACTGAAGGGAGCTCCC	NC_029680.1
44	rco-miR156c	TGACAGAAGAGAGTGAGCACA	NW_015453542.1
45	rco-miR156g	TTGACAGAAGATAGAGAGCAC	NC_029683.1
46	rco-miR156h	TTGACAGAAGATAGAGAGCAC	NC_029683.1
47	rco-miR167c	TGAAGCTGCCAGCATGATCTGG	NC_029680.1
48	rco-miR319a	TTGGACTGAAGGGAGCTCCCT	NC_029689.1
49	stu-miR156g-5p	TGACAGAAGAGAGTGAGCAC	NC_029686.1
50	tcc-miR156b	TGACAGAAGAGAGTGAGCAC	NW_015453542.1
51	tcc-miR156e	TTGACAGAAGATAGAGAGCAC	NC_029683.1
52	vun-miR162	TCGATAAACCTCTGCATCCAG	NC_029683.1
53	vun-miR319a	TTGGACTGAAGGGAGCTCCCT	NC_029689.1
54	vvi-miR156g	TTGACAGAAGATAGAGAGCAC	NC_029683.1
55	vvi-miR156i	TTGACAGAAGATAGAGAGCAC	NC_029682.1
56	vvi-miR319c	TTGGACTGAAGGGAGCTCCCT	NC_029689.1
57	vvi-miR319f	TTGGACTGAAGGGAGCTCCCT	NC_029680.1
58	novel_miR_1	ucuuuccuaccccucccauacc	NW_015454295.1
59	novel_miR_100	uauuggauuucuuucuuug	NC_029682.1
60	novel_miR_101	ucuuuccuaccccucccauacc	NC_029681.1
61	novel_miR_103	gacgaacuguauuuaugaauaugc	NC_029684.1
62	novel_miR_104	accgauccgugagcucagcuug	NC_029688.1
63	novel_miR_106	aaauuggcugauauuaug	NC_029690.1
64	novel_miR_107	gagagaauuguaaggguuuugaga	NW_015453660.1
65	novel_miR_108	ucuuuccuauuccucccauucc	NC_029681.1
66	novel_miR_11	uggaucuuguuugacagcacu	NC_029682.1
67	novel_miR_110	uauuuugugcugauuucaug	NC_029680.1
68	novel_miR_111	ugucauguggcgugcuuauuuaau	NC_029686.1
69	novel_miR_112	aagguacucuguaaaaacg	NC_029685.1
70	novel_miR_113	uuaccgauuccccccauuccc	NC_029689.1
71	novel_miR_114	uggaucuuguuugacagcacu	NC_029682.1
72	novel_miR_119	acagauauggucgagggg	NW_015453444.1
73	novel_miR_12	acuuccuucugcuaagcuaucacc	NC_029687.1
74	novel_miR_122	uggagaagcagggcacgugc	NW_015454061.1
75	novel_miR_123	uggaucuuguucgacagcacu	NC_029682.1
76	novel_miR_124	ucuggcuugucuaugaucugcgu	NC_029681.1
77	novel_miR_13	uggaucuuguucgacagcacu	NC_029682.1
78	novel_miR_130	cgauuucugacacuuucucag	NC_029685.1
79	novel_miR_134	ugauguauggaaugaagau	NC_029683.1
80	novel_miR_135	uggaucuuguuugacagcacu	NC_029682.1
81	novel_miR_136	cgcacgcuagcggggaaccauc	NW_015453517.1
82	novel_miR_138	caggacuagaagguugggca	NC_029689.1
83	novel_miR_139	uugccgacuccacccauuccca	NC_029681.1
84	novel_miR_140	guaagacuaagcucaugagcuu	NC_029690.1
85	novel_miR_143	gaaaaagaaccguuagaca	NC_029689.1

86	novel_miR_144	cucuaucacugacauacguau	NC_029684.1
87	novel_miR_145	cauggauuguuuguaugagu	NW_015453444.1
88	novel_miR_147	uguguucucaggucgccccug	NC_029680.1
89	novel_miR_149	uugucugaauuuacaaucucg	NC_029685.1
90	novel_miR_150	aucaacuuugguguaguacgca	NW_015454225.1
91	novel_miR_152	caugugcucuagcucuccagc	NC_029679.1
92	novel_miR_153	cuugcggaccgucuguaucauagc	NW_015453655.1
93	novel_miR_155	ccugagauuuuguaagagcca	NC_029682.1
94	novel_miR_157	agagugauugucuugguucu	NC_029681.1
95	novel_miR_158	aauaggccaugaaugagga	NC_029684.1
96	novel_miR_159	ugaucuguuuaacucgaaauugac	NW_015453459.1
97	novel_miR_16	uacuuguaaagaacucaaacgug	NC_029683.1
98	novel_miR_161	uggaucuuguuugacagcacu	NC_029682.1
99	novel_miR_162	uuacagguucgagaagaggca	NC_029684.1
100	novel_miR_163	uugucugaauuuacaaucuca	NC_029685.1
101	novel_miR_164	uuacaagaguuggaauucaga	NC_029686.1
102	novel_miR_165	ccaguucauccaaacaccaug	NC_029683.1
103	novel_miR_166	uggaucuuguucgacagcacu	NC_029682.1
104	novel_miR_167	uggaucuuguucgauggcacuc	NC_029682.1
105	novel_miR_168	auagacggacuguauauauauau	NW_015453691.1
106	novel_miR_176	uguuaaugaucugacuuugaagg	NC_029680.1
107	novel_miR_177	aucuuauaaaauacugcuuca	NC_029686.1
108	novel_miR_180	cgcugccaaagaugucgccaug	NW_015454364.1
109	novel_miR_181	uuccaucucuugcacacug	NC_029686.1
110	novel_miR_183	cagaauuucuaaacaucaaaucg	NW_015453474.1
111	novel_miR_184	aaucuuucuguuucaccugaa	NC_029685.1
112	novel_miR_185	uggaucuuguucgauggcacuc	NC_029682.1
113	novel_miR_187	agucaaauugaauggaugauug	NC_029681.1
114	novel_miR_197	uggcugaauagaugaauu	NC_029688.1
115	novel_miR_198	uggagaagcagggcacgugc	NW_015454179.1
116	novel_miR_200	ucuuuccuauuccucccauucc	NW_015454792.1
117	novel_miR_204	uggacuuugcugaauacuaca	NW_015453527.1
118	novel_miR_205	uacuuguaaagaacucaaacgug	NC_029689.1
119	novel_miR_207	uuucaauugggucugaauu	NC_029681.1
120	novel_miR_210	ugguucuugaacaucaugcuga	NC_029680.1
121	novel_miR_212	acuugagacgaugaugauggcacau	NC_029684.1
122	novel_miR_213	ccgauugagcugcucuuuugacuc	NW_015454285.1
123	novel_miR_214	gguguggccugagaacauagg	NC_029688.1
124	novel_miR_215	uaauugugagaaugugugua	NC_029684.1
125	novel_miR_216	uuacagguucgagaagaggca	NC_029688.1
126	novel_miR_217	uaucugcaucuguauagcuuuc	NC_029679.1
127	novel_miR_219	ucuuuccuaccccucccauacc	NC_029681.1
128	novel_miR_220	gugccaugaacuugauccaga	NW_015453762.1
129	novel_miR_221	uuuuuguuuuuugggguug	NW_015453526.1

130	novel_miR_222	aguagagacaugguaguucaaa	NC_029682.1
131	novel_miR_225	uugaauugagacucuaaguug	NC_029687.1
132	novel_miR_229	uuuuugguacaucacauucaugc	NW_015453447.1
133	novel_miR_231	agauggauuuggagauggag	NC_029686.1
134	novel_miR_27	uggagaagcagggcacgugc	NC_029687.1
135	novel_miR_29	uuuacugggcuugggcuu	NW_015453467.1
136	novel_miR_3	gaguaguucuagaucuggucc	NW_015453449.1
137	novel_miR_30	agauggauuuggagauggag	NC_029686.1
138	novel_miR_31	uuaaguuugauugauagcu	NC_029687.1
139	novel_miR_33	uugauuguuuucauucugaua	NC_029682.1
140	novel_miR_34	uggaucuuguuugacagcacu	NC_029682.1
141	novel_miR_35	aauagaggauaaguaaacag	NC_029685.1
142	novel_miR_36	guguuaguugucauuuuagugcu	NC_029685.1
143	novel_miR_4	ugaugugcucguggaacugaacc	NC_029689.1
144	novel_miR_40	caaaugaguguggauacccagga	NC_029683.1
145	novel_miR_41	aaagaggaagaggguggug	NC_029682.1
146	novel_miR_42	uggaucuuguuugacagcacu	NW_015453762.1
147	novel_miR_43	aggaguauggaaaauugugc	NC_029684.1
148	novel_miR_44	uggaucuuguucgacagcacu	NC_029682.1
149	novel_miR_48	aaguggaaugcuguucgaucgaauc	NC_029680.1
150	novel_miR_50	uggaucuuguucgauggcacuc	NC_029682.1
151	novel_miR_53	accgauccgugagcucagcuug	NC_029688.1
152	novel_miR_54	uggaucuuguucgacagcacu	NC_029682.1
153	novel_miR_55	aguuguaguugcuugacgu	NW_015453522.1
154	novel_miR_6	ucuuuccuauuccucccauucc	NW_015454706.1
155	novel_miR_63	ucuuuccuaccccucccauacc	NW_015454792.1
156	novel_miR_64	ccgggaugauggaagaugcugagg	NC_029686.1
157	novel_miR_65	uuaaugaugccuccuuccuauagc	NC_029679.1
158	novel_miR_68	uagaauuccauaacaauccauc	NW_015454086.1
159	novel_miR_69	caucaucugugcucucuaucu	NC_029683.1
160	novel_miR_7	acuugagacgaugaugauggcacau	NW_015453582.1
161	novel_miR_70	uucuguaguuccuggaaucug	NC_029686.1
162	novel_miR_73	acgauguguguaucuagggaa	NW_015453508.1
163	novel_miR_75	acggugaagaaaaauguuuuu	NC_029682.1
164	novel_miR_76	ucacuaauacugcgguucucaugc	NC_029689.1
165	novel_miR_77	ucuuuccuaccccucccauacc	NW_015454706.1
166	novel_miR_78	ugcuaacuuguagacguugugagg	NC_029682.1
167	novel_miR_79	uuagauucacgcacaaacucg	NC_029683.1
168	novel_miR_8	cagggaagcggcagagcaugg	NC_029687.1
169	novel_miR_80	auuucguuguauagaauggcgug	NC_029690.1
170	novel_miR_82	ucuuuccuauuccucccauucc	NW_015454295.1
171	novel_miR_83	ucauauuuguuccaucgccugugg	NC_029680.1
172	novel_miR_87	caaauaaugaucuacguaua	NW_015453496.1
173	novel_miR_88	uggagaagcagggcacgugc	NC_029680.1

174	novel_miR_92	uggaucuuguuugacagcacu	NC_029682.1
175	novel_miR_95	uuugugucuuuccuuugua	NC_029682.1
176	novel_miR_96	uauuuaacaacucuuugaauguag	NC_029685.1
177	novel_miR_98	guaagacuaagcucaugagcuu	NC_029690.1
